# Our successful surgical approach in a giant vegetative mass diagnosed on aortic valve

**DOI:** 10.1186/1749-8090-8-S1-P14

**Published:** 2013-09-11

**Authors:** A Gurbuz, U Yetkin, I Yurekli, I Peker

**Affiliations:** 1 Izmir Katip Celebi University Ataturk Training and Research Hospital, Department of Cardiovascular Surgery, Turkey

## Background

Cardiac complications of infective endocarditis occur via vegetations on heart valves.

## Method

Our case was a 46-year-old female. She was suffering from shortness of breath for one week increasing in intensity. Transesophageal echocardiography revealed moderate aortic regurgitation and a hypermobile mass of 15x20 mm with hyper- and hypoechoic densities sitting on all leaflets of the aortic valve moving in and out of the left ventricular outflow tract. She was hospitalized for an emergency operation. Her past medical history was significant for a severe upper respiratory tract infection 4 months ago.

## Results

After a median sternotomy and standard cannulation, an aortotomy incision was done. A giant vegetative mass was expanding from beneath non-coronary cusp to the anterior leaflet of mitral valve, probably due to late endocarditis. This broad based vegetation was totally extirpated. Due to severe destruction of the aortic valve, it was completely excised. Then, valve replacement with 21 mm St. Jude bioprosthetic valve was performed. Early postoperative period was event-free. After consulting with Department of Infectious Diseases she was discharged with oral antibiotic regimen. Her late postoperative outpatient follow-up continues.

## Conclusion

Intracardiac injury develops more severely if the diagnosis of native valve endocarditis is delayed or if the etiological agents are more virulent and resistant to antibiotherapy. Surgical therapy becomes more widely used. Surgery may even be required in 20-40% of patients after recovery from infective endocarditis.

